# Development and external validation of a nomogram with inflammatory markers for predicting invasiveness of intraductal papillary mucinous neoplasm of pancreas

**DOI:** 10.1097/MD.0000000000029036

**Published:** 2022-03-18

**Authors:** So Jeong Yoon, Hongbeom Kim, Okjoo Lee, Ji Hye Jung, Chang-Sup Lim, Yong Chan Shin, Wooil Kwon, Jin-Young Jang, Sang Hyun Shin, Jin Seok Heo, In Woong Han

**Affiliations:** ^a^ *Division of Hepatobiliary-Pancreatic Surgery, Department of Surgery, Samsung Medical Center, Sungkyunkwan University School of Medicine, Seoul, South Korea,* ^b^ *Division of Hepatobiliary and Pancreatic Surgery, Department of Surgery, Seoul National University Hospital, Seoul National University College of Medicine, Seoul,* *South Korea,* ^c^ *Department of Surgery, Seoul Metropolitan Government - Seoul National University Boramae Medical Center, Seoul National University College of* *Medicine, Seoul, South Korea,* ^d^ *Department of Surgery, Ilsan Paik Hospital, Inje University College of Medicine, Goyang, South Korea.*

**Keywords:** inflammatory markers, intraductal papillary mucinous neoplasm, malignancy risk, nomogram, pancreatic cancer

## Abstract

Recent studies have reported that inflammatory markers, such as neutrophil-to-lymphocyte ratio, platelet-to-lymphocyte ratio, and advanced lung cancer inflammation index, are associated with invasiveness of intraductal papillary mucinous neoplasm (IPMN). This study aimed to develop and validate a new nomogram that includes inflammatory markers for predicting the invasiveness of IPMN.

The data of 365 patients who underwent surgical resection for IPMN at 4 centers between 1995 and 2016 were retrospectively reviewed to develop a new nomogram. For external validation, a separate patient cohort was used. The predictive ability of the nomogram was evaluated using the area under the receiver operating characteristic curve.

The new nomogram was developed using the following variables which were identified as risk factors for invasive IPMN: body mass index, preoperative serum bilirubin level, carbohydrate antigen 19-9, neutrophil-to-lymphocyte ratio, platelet-to-lymphocyte ratio, advanced lung cancer inflammation index, main duct type, presence of solid portion, and tumor size. After external validation, the area under the curve value was 0.649 (95% CI: 0.578-0.720, *P* < .001).

To the best of our knowledge, this study is the first to predict and externally validate the invasiveness in IPMN using inflammatory markers. Further research is necessary to improve predictability of the model for selecting patients for surgical resection.

## 1. Introduction

Intraductal papillary mucinous neoplasms (IPMNs) of the pancreas are cystic neoplasms that have a broad histological spectrum ranging from benign to malignant disease. The incidence has been increasing over time as advanced imaging tests facilitate the recognition of pancreatic cystic lesions.^[1]^ From histopathological point of view, IPMN is often mentioned together with mucinous cystic neoplasm, since both have malignant potential. IPMNs can be distinguished from mucinous cystic neoplasms by the presence or absence of communication with the pancreatic duct.^[2,3]^

Prediction of malignant IPMN based on radiologic features has become feasible using magnetic resonance cholangiopancreatography scan or endoscopic ultrasound guided biopsy. According to the recently revised consensus guidelines of the International Association of Pancreatology (IAP),^[4]^ “high-risk stigmata,” which suggests malignancy of branch duct intraductal papillary mucinous neoplasm (BD-IPMN), includes measurement of the enhanced mural nodule and the size of the main pancreatic duct in the imaging tests. Several studies have attempted to invent nomograms for risk analysis including the imaging features, and reported variable predictive values.^[5-8]^

However, in addition to image findings that directly reflect the characteristics of the lesions, using other factors that are associated with malignant IPMN would be useful to develop a more accurate quantitative scoring model. Previous studies have shown that inflammatory markers, such as neutrophil-tolymphocyte ratio (NLR) and platelet-to-lymphocyte ratio (PLR) are associated with poor survival rates in patients with pancreatic cancer and other malignancies.^[9-11]^ These markers were identified as useful predictors of malignant potential in IPMN.^[12-15]^

The purpose of this study is to develop a newly expanded nomogram by including the inflammatory markers, that quantifies the risk of malignancy in IPMN patients. We also validated the predictability of the new model using external data.

## 2. Methods

### 2.1. Data collection

The cohort for developing a nomogram included patients who were diagnosed with IPMN based on pathologic examination after surgical resection, between April 1995 and December 2016 at 4 different centers: Samsung Medical Center (SMC), Seoul National University Boramae Medical Center (BRM), Ilsan Paik Hospital (IPH), and Dongguk University Ilsan Hospital (DUH). The pathologic diagnosis and review of IPMN was done by each hospital’s pathologists according to the 4th edition of WHO classification.^[16]^

Among 468 patients, 103 patients were excluded for the following reasons: history of pancreatitis, history of cholangitis or other malignancy, or absence of data regarding carbohydrate antigen 19-9 (CA19-9). Finally, a total of 365 patients were included in the analyses. This study was approved by the Institutional Review Board of Samsung Medical Center (Seoul, Korea, the approval number: 2017-07-016).

### 2.2. Clinical variables for analysis

The patients’ demographics, preoperative laboratory results, and image findings were retrospectively reviewed. Blood laboratory tests were performed at least once within a month before surgery, and the results of the most recent examinations were used in the analysis. NLR was calculated as the total count of neutrophils divided by the total count of lymphocytes and PLR as the total count of platelets divided by the total count of lymphocytes. Advanced lung cancer inflammation index (ALI) was calculated as follows: body mass index (BMI) × serum Albumin/NLR. The values that were required for the calculations were measured before the operations. The radiological assessments including computed tomography, magnetic resonance imaging, endoscopic ultrasound were conducted preoperatively with a focus on the type and size of IPMN, as well as the presence of solid portion and the size of the main pancreatic duct. The sizes of tumor and pancreatic duct were defined as the largest diameter of the main mass and the main pancreatic duct, respectively. In case of patients with >2 preoperative images by different modalities, the largest figures were adopted. The type of IPMN was classified as branch duct type, main duct type, or mixed type in accordance with the 2017 revised Fukuoka guidelines.^[4]^ After surgical resection, pathological analysis and histological grading were performed according to the criteria of the WHO classification system for digestive neoplasms. This classification system includes low or intermediate dysplasia, high-grade dysplasia, and IPMN with invasive cancer.^[16]^

### 2.3. Development of nomogram

In our previous study,^[15]^ a comparative analysis was performed between the patients with non-invasive and invasive IPMN in the development cohort. After the risk factor analysis for invasive IPMN, we selected variables for a new nomogram.

A nomogram was built after fitting in the regression model. First, we used the distribution summaries of the included variables with plotting ranges and values to be adjusted. Using the linear predictor method, the factor with the greatest impact was determined and the other factors were assigned in consecutive order. All the predictors with assigned values were mapped in the “Point” axis. To estimate the predictability of the new nomogram, internal validation was conducted using bootstrap resamples.

### 2.4. External validation

For external validation, we extracted another patient cohort from Seoul National University Hospital (SNUH), which included 384 pathologically-proven IPMN patients after surgical resection between May 1995 and December 2016. The pathology reports were reviewed according to the 4th edition of WHO classification.^[14]^ The same exclusion criteria used for the development set were applied to the external validation set. The new receiver operating characteristic curve was drawn to verify the predictability of the model.

The development of the nomogram and its internal and external validation were conducted using R 3.4.3 (Vienna, Austria).

## 3. Results

The demographic and clinical data from the development set (n = 365) are summarized in Table 1. The median age of the patients was 63 years and 66.3% were men. The median values of NLR, PLR, and ALI were 1.8, 109.5, and 56.3, respectively. The most common type of IPMN was BD-IPMN (50.7%). The solid portion in the preoperative imaging was found in 89 (24.4%) patients. The median size of tumor and duct was 3.1cm and 4.7mm, respectively. Among these patients, 98 (26.8%) patients demonstrated the presence of invasive IPMN in the surgical pathology.

**Table 1:** Demographic and clinical data of the development set (n = 365).

**Variables**		**N (%) mean or median (IQR)**
Age, median, yrs		63 (57-69)
Sex, male		242 (66.3)
BMI, median, kg/m^2^		23.8 (22.1-25.5)
Maximum pre-op serum bilirubin, mean, mg/dL		0.7 (0.5-1.2)
CA 19-9, median, U/mL		12.7 (6.4-24.7)
NLR, median		1.8 (1.3-2.5)
PLR, median		109.5 (85.7-152.4)
ALI, median		56.3 (36.8-80.6)
Type of IPMN, n (%)	
	Main duct type	76 (20.8)
	Branch duct type	185 (50.7)
	Mixed type	104 (28.5)
Tumor size, mean, cm		3.1 (2.4-4.2)
Pancreatic duct size, mean, mm		4.7 (3.0-7.0)
Solid portion^†^, n (%)		89 (24.4)
Operation, n (%)	
	Pancreaticoduodenectomy	184 (50.4)
	Distal pancreatectomy	120 (32.9)
	Central pancreatectomy	13 (3.6)
	Others	48 (13.1)
Pathology, n (%)	
	Non-invasive^*^	267 (73.2)
	Invasive	98 (26.8)
ALI = advanced lung cancer inflammation index, BMI = body mass index, CA 19-9 = carbohydrate antigen 19-9, IPMN = intraductal papillary mucinous neoplasm, IQR = interquartile range, NLR = neutrophil-to-lymphocyte ratio, PLR = platelet-to-lymphocyte ratio, Pre-op = preoperative.		

^*^ Low, intermediate or high-grade dysplasia.

^†^ Solid portion, mural nodule, or enhanced cyst.

Based on our previous study, the following variables which were identified as significant were included in developing the nomogram^[13]^: NLR, PLR, ALI, maximum preoperative serum bilirubin level, the solid portion of the tumor, BMI, size of the tumor, carbohydrate antigen 19-9, and type of IPMN. A draft of the new nomogram is shown in Fig. 1. To obtain a total point, points from the point axis for each predictor were added together. The individual risk for invasive IPMN can be obtained using the axis of “predicted value.”

**Figure:**
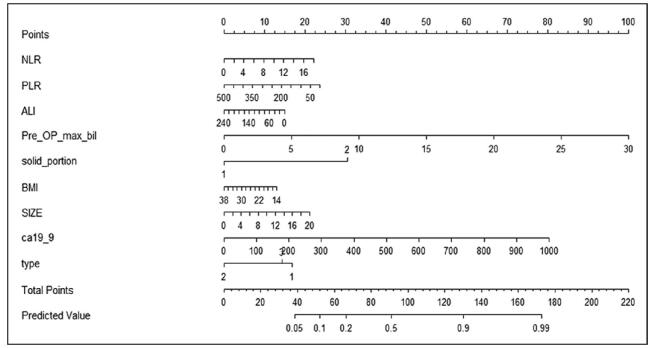
**Figure 1.** The new nomogram predicting risk the of malignancy in IPMN. IPMN = intraductal papillary mucinous neoplasm.

To internally validate the predictive performance of the model, resampling with a thousand bootstrap samples was done (Fig. 2). In the figure, the *x*-axis is the predicted probability and the *y*-axis is the actual probability of invasive IPMN. The dashed line indicates the relationship between the actual and predicted probabilities when prediction is perfect. The dotted line was drawn using the new nomogram and very close to the ideal line, with the mean absolute error of 0.025, which means good calibration. Based on the results, we created a web calculator that can be easily used to predict the malignancy risk of IPMN by entering clinical data (http://ipmn.smchbp.org/) (Fig. 3).

**Figure:**
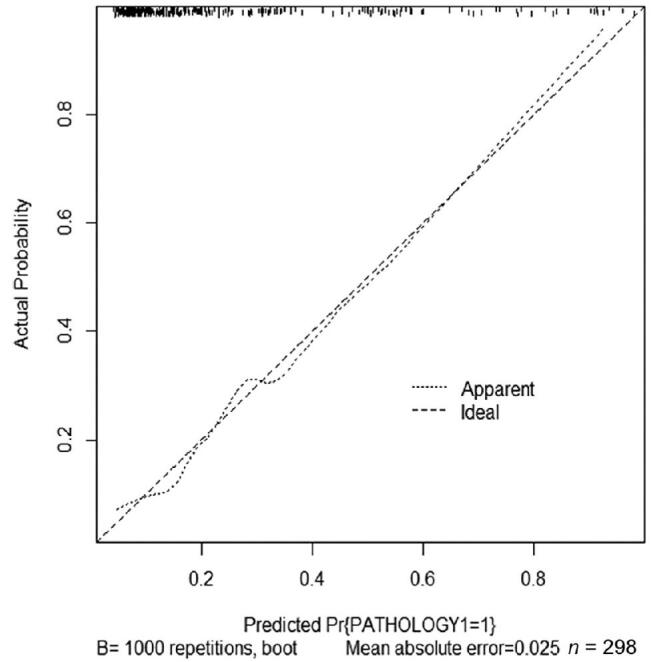
**Figure 2.** Calibration plot of internal validation through 1000 bootstrap resamples.

**Figure:**
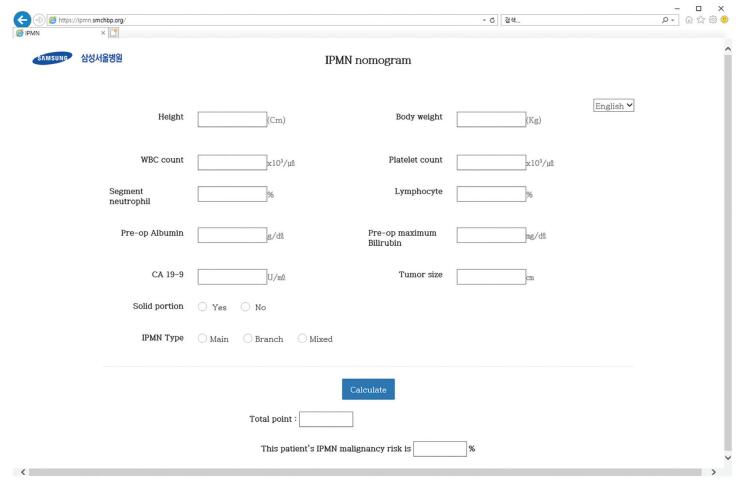
**Figure 3.** The website for calculating malignancy risk of IPMN. IPMN = intraductal papillary mucinous neoplasm.

Table 2 summarizes the demographic and clinical features of the external validation set (n = 384). The median values of NLR, PLR, and ALI were 1.8, 114.5, and 54.3, respectively. The mean tumor size was 3.5cm and the mean diameter of the pancreatic duct was 6.0 mm. Among the patients in the validation cohort, BD-IPMN was identified as the most common type (n = 185, 55.7%), and the solid portion was found in 99 (25.8%) patients. Based on the pathology reports, 102 (25.3%) patients were identified to have invasive IPMN. After excluding the patients with missing variables, a total of 308 patients were included in external validation. The receiver operating characteristic curve is shown in Fig. 4, and the area under the curve (AUC) of the nomogram was identified as 0.649 (95% CI: 0.578-0.720, *P* < .001).

**Table 2:** Demographic and clinical data of the external validation set (n = 384).

**Variables**		**N (%) mean or median (IQR)**
Age, median, yrs		65 (59-70)
Sex, male 219		(57.0)
BMI, median, kg/m^2^		23.2 (20.9-25.1)
Maximum pre-op serum bilirubin, mean, mg/dL		1.0 (0.6-1.1)
CA 19-9, median, U/mL		10.1 (5.0-24.2)
NLR, median		1.8 (1.3-2.3)
PLR, median		114.5 (89.4-149.1)
ALI, median		54.3 (36.0-74.9.6)
Type of IPMN, n (%)	
	Main duct type	38 (9.9)
	Branch duct type	214 (55.7)
	Mixed type 132 (34.4)
Tumor size, mean, cm		3.5 (2.1-4.0)
Pancreatic duct size, mean, mm		6.0 (2.3-8.0)
Solid portion^†^, n (%)		99 (25.8)
Operation, n (%)	
	Pancreaticoduodenectomy	192 (50.0)
	Distal pancreatectomy	122 (31.8)
	Central pancreatectomy	17 (4.4)
	Others	53 (13.8)
Pathology, n (%)	
	Non-invasive^*^	282 (74.7)
	Invasive	102 (25.3)
ALI = advanced lung cancer inflammation index, BMI = body mass index, CA 19-9 = carbohydrate antigen 19-9, IPMN = intraductal papillary mucinous neoplasm, IQR = interquartile range, NLR = neutrophil-to-lymphocyte ratio, PLR = platelet-to-lymphocyte ratio, Pre-op = preoperative.		

^*^ Low, intermediate or high-grade dysplasia.

^†^ Solid portion, mural nodule or enhanced cyst.

**Figure:**
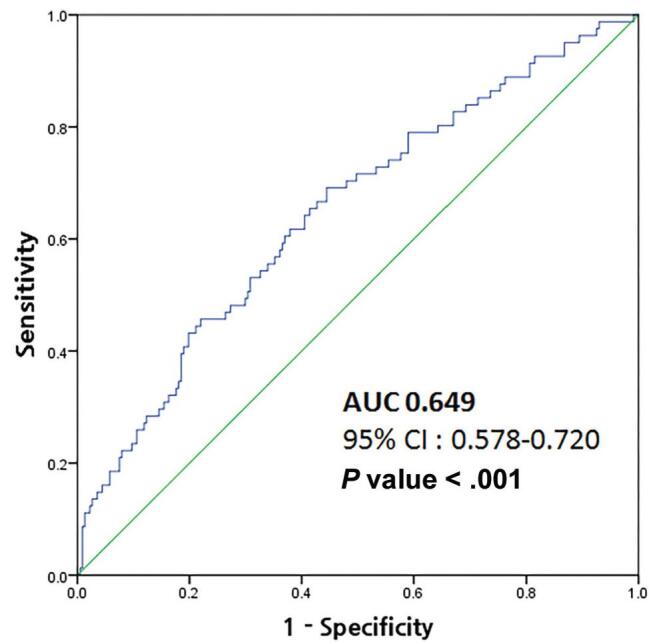
**Figure 4.** The receiver operating curve (ROC) of the new nomogram.

## 4. Discussion

To the best of our knowledge, this is the first study to propose a nomogram with inflammatory markers for predicting invasiveness in patients with IPMN. The predictive power of the nomogram demonstrated in the external validation was acceptable (AUC: 0.649, 95% CI: 0.578-0.720, *P* < .001). Nomograms are considered useful as they provide a graphical interface for interpreting the effect sizes of predictors in multivariable models. In the present study, we have also established a user-friendly web site for calculation.

Since IPMNs are known to have malignant potential, it is clinically important to determine surgical candidates by predicting the invasiveness of IPMN preoperatively. In addition, the fact that IPMN mostly occurs in older patients raises the need for more sophisticated surgical indications to minimize unnecessary surgery.^[17]^ The International consensus guidelines, which are regarded as the most reliable resources, have been updated based on cytology and image findings to predict malignancy and present a flow chart for surveillance of IPMN.^[4]^ However, several studies have argued that the positive predictive value of the guideline remains limited (27-62%).^[18-20]^ Among these, one study evaluated the utility of the current guidelines and identified 40 (10.5%) patients with malignancy among 382 low-risk patients.^[18]^ This finding suggests that the risk of malignancy may be underestimated based on the guidelines alone and further research for identifying potential risk factors is needed.

Several studies have shown the clinical significance of NLR and PRL in predicting the prognosis of pancreas ductal adenocarcinoma,^[9,21]^ as well as other solid tumors.^[10,11,22]^ In a metaanalysis that assessed the prognostic ability of NLR on overall survival in patients with pancreatic cancer,^[23]^ a high preoperative NLR was identified to be associated with a poorer prognosis than that of patients with lower NLR values, with the cut-off values ranging from 2 to 5. In pancreas ductal adenocarcinoma initiation, neutrophil activity and the release of inflammatory mediators may play central roles in mediating peritumoral inflammation and failed protective immunity, which results in decreased lymphocyte counts.^[24]^ Following this, the association of tumor-associated neutrophils (TANs) with malignant progression in IPMNs was identified.^[25]^ Another study showed that NLR was significantly higher in malignant IPMN patients compared with non-invasive IPMN.^[26]^ The authors also found that NLR in the non-invasive IPMN group was higher than in healthy patients. In addition, NLR decreased after surgical resection in both invasive and non-invasive IPMN groups. Likewise, in our previous study, we identified NLR as an independent prognostic factor of malignant IPMN. The higher recurrence rate of invasive IPMN in patients with NLR ≥3.5 supports a close association between NLR and invasiveness of IPMN.^[15]^

In terms of ALI, some studies reported that low ALI could serve as a predictor for worse outcomes in other malignancies.^[27,28]^ Also, BMI and serum albumin level, which are the components of ALI, have been studied as prognostic factors for pancreatic cancer.^[29,30]^ We hypothesized that ALI might be useful in identifying high-risk IPMNs, considering that BMI and NLR were also related to the invasiveness of IPMN. There exist only limited data on the association between ALI and invasive IPMN, and there have been studies investigating the new predictive factors such as cystic fluid inflammatory markers.^[31,32]^ Apparently, there remains significant room for more research to validate the roles of these factors, and to develop another platform with improved predictability including the valid factors.

The present study has several limitations, including a limited number of patients and the retrospective design. Above all, the AUC was 0.649, which was below the reported values from previous nomogram studies (0.747-0.787).^[6,7]^ Shimizu et al^[6]^ suggested a simple nomogram with only 4 factors: sex, type of lesion, size of mural nodules, and cytology of pancreatic juice. Following that, another nomogram with 6 variables including carbohydrate antigen 19-9 was proposed in a cross-national multicenter study.^[7]^ In spite of adding inflammatory markers, our new developed nomogram showed a relatively lower AUC. This might be mainly due to heterogeneity of data, caused by individual hospital’s different diagnostic facilities and faculty members who reviewed the clinical data. The study may also have been subject to selection bias, as we only included patients who underwent surgical resection and did not consider the patients who were on active surveillance. Nonetheless, this research was the first to attempt to include inflammatory markers in the predictive platform for invasive IPMN, and it could be the groundwork for future studies. A nationwide prospective study that includes molecular biology with the combination of artificial intelligence techniques is under consideration at our institution to improve the accuracy and predictive value of our risk assessment models.

In conclusion, we have developed a nomogram with inflammatory markers which are recently investigated predictors of invasive IPMN. The results of the external validation leave considerable room for improvement. Further extensive research using different methodology is needed to more accurately predict the risk of malignancy in IPMN.

## Acknowledgment

The authors would like to thank Hyemin Kim (Data Manager, Department of Surgery, Samsung Medical Center, Sungkyunkwan University School of Medicine) for help with data collection.

## Author contributions

(I) Conception and design: So Jeong Yoon, Hongbeom Kim, Chang-Sup Lim, Yong Chan Shin, Wooil Kwon, Jin-Young Jang, Sang Hyun Shin, Jin Seok Heo, In Woong Han; (II) Administrative support: Jin-Young Jang, In Woong Han; (III) Provision of study materials or patients: So Jeong Yoon, Hongbeom Kim, Chang-Sup Lim, Yong Chan Shin, Wooil Kwon, Jin-Young Jang, Sang Hyun Shin, Jin Seok Heo, In Woong Han; (IV) Collection and assembly of data: So Jeong Yoon, Hongbeom Kim, Chang-Sup Lim, Yong Chan Shin, Wooil Kwon, Jin-Young Jang, Sang Hyun Shin, Jin Seok Heo, In Woong Han; (V) Data analysis and interpretation: all authors; (VI) Manuscript writing: all authors; (VII) Final approval of manuscript: all authors
